# Complete loss of PAX4 causes transient neonatal diabetes in humans

**DOI:** 10.1016/j.molmet.2025.102201

**Published:** 2025-07-02

**Authors:** James Russ-Silsby, Yunkyeong Lee, Varsha Rajesh, Mahsa Amoli, Nasser Ali Mirhosseini, Tushar Godbole, Matthew B. Johnson, D. Evelyn Ibarra, Han Sun, Nicole A.J. Krentz, Matthew N. Wakeling, Sarah E. Flanagan, Andrew T. Hattersley, Anna L. Gloyn, Elisa De Franco

**Affiliations:** 1 Department of Clinical and Biomedical Sciences, Faculty of Health and Life Sciences, University of Exeter, Exeter, UK; 2 Division of Endocrinology, Department of Pediatrics, School of Medicine, Stanford University, Stanford, CA, USA; 3 Metabolic Disorders Research Center, Endocrinology and Metabolism Molecular Cellular Sciences Institute, Tehran University of Medical Sciences, Tehran, Iran; 4 Department of Pediatrics, Shahid Sadoughi University of Medical Sciences, Yazd, Iran; 5 Dr. Vasantrao Pawar Medical College, Nashik, India; 6 Department of Genetics, School of Medicine, Stanford University, Stanford, CA, USA; 7 Stanford Diabetes Research Center, School of Medicine, Stanford University, Stanford, CA, USA

**Keywords:** Neonatal diabetes, Monogenic diabetes, PAX4, Pancreatic beta cell development

## Abstract

**Objective:**

Gene discovery studies in individuals with diabetes diagnosed within 6 months of life (neonatal diabetes, NDM) can provide unique insights into the development and function of human pancreatic beta-cells.

**Methods:**

We performed genome sequencing in a cohort of 43 consanguineous individuals with NDM in whom all the known genetic causes had previously been excluded. We used quantitative PCR and RNA-sequencing in CRISPR-edited human induced pluripotent stem cells (iPSCs), and CUT&RUN-sequencing in EndoC-βH1 cells to investigate the effect of *PAX4* loss on human pancreatic development.

**Results:**

We describe the identification of homozygous *PAX4* loss-of-function variants in 2 individuals with transient NDM: a p.(Arg126∗) stop-gain variant and a c.-352_104del deletion affecting the first 4 *PAX4* exons. We confirmed the p.(Arg126∗) variant causes nonsense mediated decay in CRISPR-edited iPSC-derived pancreatic endoderm cells. Integrated analysis of CUT&RUN-sequencing in EndoC-βH1 cells and RNA-sequencing in *PAX4*-depleted islet stem cell models identified genes directly regulated by PAX4 involved in both pancreatic islet development and glucose-stimulated insulin secretion.

**Conclusion:**

We report the first human cases of complete loss of *PAX4*, establishing it as a novel cause of NDM and highlighting its role in human beta cell development. Both probands had transient NDM which remitted in early infancy but relapsed at the ages of 2.4 and 6.7 years, demonstrating that in contrast to mouse models, PAX4 is not essential for the development of human pancreatic beta-cells.

## Highlights

• Homozygous loss-of-function variants in *PAX4* are a novel genetic cause of transient neonatal diabetes.
• PAX4 directly regulates genes involved in pancreatic beta cell development and glucose-sensitive insulin secretion.
• The role of PAX4 in humans differs to that observed in mouse and is not essential for beta cell development.

## 1 Introduction

The identification of genes in which bi-allelic loss-of-function (LoF) variants cause diabetes onset in the first 6 months of life (neonatal diabetes mellitus, NDM) can provide unique insights into the development and function of human pancreatic beta cells. Historically, animal gene knockout models, in particular mice, have been used to study pancreatic development. However, many differences exist between human and murine pancreatic development, including the transcription factors which control the process [1]. Biallelic LoF variants naturally mimic the functional consequences of gene knockouts examined in animal models. Thus, by studying individuals with recessive NDM, we can identify human-specific effects of losing genes involved in pancreatic development [[2], [3], [4]]. An example of this is the recent identification of biallelic LoF variants in *ZNF808* as a cause of NDM and pancreatic agenesis [5]. *ZNF808* is a primate-specific gene which is absent in all other mammals, including rodents. Through gene discovery in NDM, it has been demonstrated to have a critical role in pancreatic cell fate specification during human development.

In this study we use genome sequencing to identify homozygous *PAX4* LoF variants as a novel cause of NDM and functionally explore the human role of the gene using Cleavage Under Targets and Release Using Nuclease (CUT&RUN) [6] and RNA sequencing in human beta cell models.

## 2 Results and discussion

### 2.1 Homozygous *PAX4* LoF variants are a novel cause of transient neonatal diabetes

**Figure 1:**
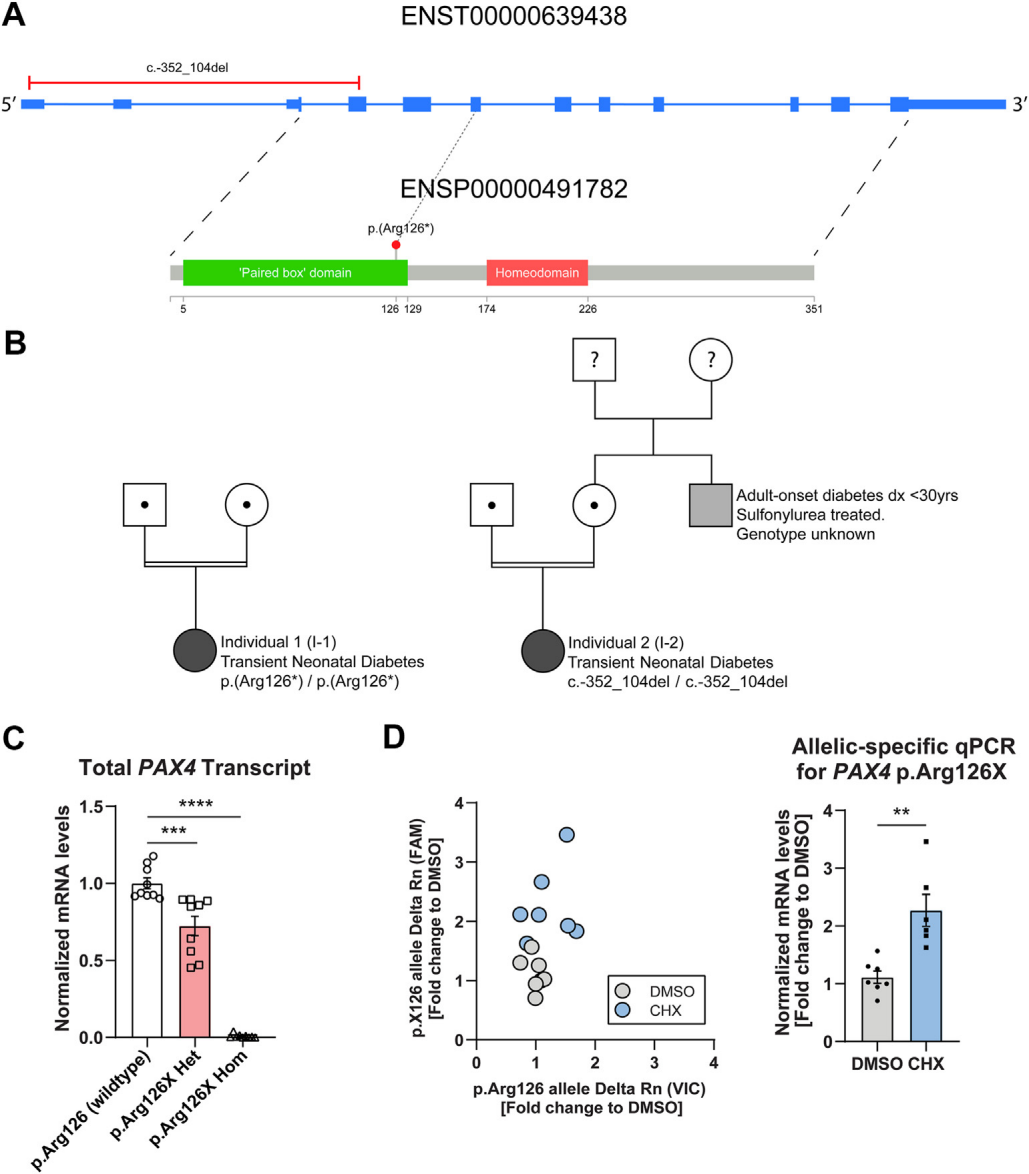
A) Two homozygous *PAX4* loss-of-function (LoF) variants were identified in two unrelated individuals. The variants are both predicted to cause complete ablation of PAX4 translation. B) The two individuals (filled black circle) had transient neonatal diabetes and were born to consanguineous parents. No additional individuals with TNDM were reported in either family. C) Reduction in *PAX4* transcript in hiPSC-PE harboring p. (Arg126∗) variant. A mean ∼27.6 % reduction in total *PAX4* transcript expression was observed in the p.(Arg126∗) heterozygous samples and a mean ∼99.1 % reduction in the p.(Arg126∗) homozygous samples (three independent clones performed in triplicate, respectively) when compared to the wildtype samples (three independent clones performed in triplicate). D) Allele-specific qPCR for the *PAX4* transcript in p.Arg126X heterozygous cells following 100 μg/ml cycloheximide (CHX) treatment for 4 h. Data are presented as mean ± SEM. Statistical analyses were performed by unpaired *t*-test or one-way ANOVA. ∗*p* < 0.05, ∗∗*p* < 0.01, ∗∗∗*p* < 0.01, ∗∗∗∗*p* < 0.0001.

Through genome sequencing, we identified homozygous LoF variants in the pancreatic homeobox transcription factor gene *PAX4* in two individuals (Figure 1A,B). Both were diagnosed with transient neonatal diabetes (TNDM), a subtype of NDM that is characterised by a period of remission in infancy and childhood where no diabetes treatment is required. No other genes containing homozygous LoF variants were identified in more than one individual in the consanguineous NDM study cohort (*N* = 43; 19 with TNDM), within whom all known genetic causes of NDM had previously been excluded through comprehensive genetic testing of the known NDM etiologies [7,8]. *PAX4* has long been considered a strong candidate gene for NDM due to its observed role in mammalian beta cell development [[9], [10], [11], [12]] and its known association with type 2 diabetes [13]. The homozygous *PAX4* LoF variants identified were a p.(Arg126∗) stop gain in individual 1 (I-1) and a 2.65 kb deletion on chromosome 7 (c.-352_104del; GRCh37(chr7): g.127255495_127258142del) in individual 2 (I-2). The stop gain variant was heterozygous in 9 individuals in the gnomAD v4 database [14], which contains single variant and indel exonic data for 807,162 individuals, giving it an allele frequency of 5.56 × 10^−6^. The deletion was not present in the gnomAD v4 structural variant (SV) dataset that contains genome-wide structural variant data for 63,046 individuals. No high-confidence homozygous *PAX4* LoF variants were present in gnomAD v4, supporting biallelic loss-of-function variants being disease-causing. Both variants were predicted to result in complete loss of *PAX4* mRNA: the stop gain variant p.(Arg126∗) is located in the 6th of 12 exons of the Matched Annotation from NCBI and EMBL-EBI (MANE) select *PAX4* transcript (ENST00000639438) and is therefore predicted to result in mRNA degradation through nonsense-mediated decay (NMD), while the 2.65 kb deletion (c.-352_104del) identified in I-2 removes the first 4 exons of the gene and its promoter.

We investigated the impact of the p.(Arg126∗) variant on *PAX4* transcript levels by generating CRISPR-edited human induced pluripotent stem cells (iPSCs) harboring heterozygous and homozygous p.(Arg126∗) variants. These iPSCs were differentiated into pancreatic endoderm (PE) cells, a stage where *PAX4* is abundantly expressed, following the protocol described by Rezania et al. [15]. Total *PAX4* expression was then compared across isogenic controls (CRISPR-sham), heterozygous, and homozygous p.(Arg126∗) cell lines. *PAX4* p.(Arg126∗) heterozygous cells had a 27.6% reduction and homozygous cells had a 99.1% reduction in transcript levels (Figure 1C). Nonsense-mediated decay (NMD) serves as a quality control process that eliminates mRNAs containing premature stop codons, thereby preventing the production of incomplete or aberrant proteins [16]. To assess whether the p.(Arg126∗) variant leads to NMD of the *PAX4* transcript, we performed allele-specific qPCR following cycloheximide (CHX) treatment in iPSC-PE cells heterozygous for the p.(Arg126∗) variant. CHX is a known NMD inhibitor that stabilizes transcripts containing premature stop codons. We measured the relative abundance of the wildtype (p.Arg126) and mutant (p.Arg126∗) alleles. Upon CHX treatment, we observed a significant increase in the mutant allele transcript, whereas the wild-type allele remained unchanged (Figure 1D). This result supports our hypothesis that the p.(Arg126∗) variant leads to selective degradation of the mutant transcript through NMD, contributing to the observed reduction in total *PAX4* expression in heterozygous and homozygous lines.

We next investigated the presence of autosomal recessive *PAX4* variants in a replication cohort of 6,087 individuals with suspected monogenic diabetes using a combination of targeted next generation sequencing (tNGS) and genome sequencing. No additional homozygous or compound heterozygous *PAX4* LoF variants were identified. This replication cohort included 476 individuals with NDM diagnosed before 6 months and 5,611 individuals with clinically suspected monogenic diabetes (age at diagnosed range: 6 months to 60 years). 383 individuals were born to consanguineous parents. The absence of biallelic LoF variants in the replication cohort suggests that loss of *PAX4* is a rare cause of monogenic diabetes.

The two individuals with homozygous *PAX4* LoF variants had similar clinical features (Supplemental Table 1). Both were diagnosed with diabetes after the first month of life: at 6.5 weeks for I-1 and 9 weeks for I-2. Diabetes remission occurred at 7 and 8 months of age and relapsed at 2.4 and 6.7 years, respectively. Age at diabetes onset and remission are similar to those observed in individuals with TNDM caused by activating pathogenic variants in the K_ATP_ channel genes *ABCC8* and *KCNJ11*, which has a median age at onset of 4 weeks (range: 0–16 weeks) [17] and a median age upon entering remission of 8 months [17]. The ages at relapse are also consistent with the relapse range for K_ATP_-related TNDM, which typically occurs between 3 and 15 years (median: 4.7 years) [17]. Both children with *PAX4*-TNDM were treated with insulin at diagnosis and upon relapse (dose at last assessment for I-1 1U/Kg/day, dose not available for I-2). They also had reduced birth weights for their gestational age at −1.16 SD and −2.98 SD, consistent with reduced *in utero* insulin secretion during the third trimester, a critical period in foetal growth where insulin serves as the primary growth factor [18]. No extra pancreatic features were reported in either patient.

The parents of the two probands, who are heterozygous carriers of the respective variants, did not have diabetes at the time of recruitment. However, the parents of the proband with the p.(Arg126∗) variant were both under the age of 35 (34 and 32 years, the age of the parents was not recorded for the second individual). This is younger than the age at diabetes onset for two of three individuals heterozygous for the previously reported p.(Tyr186∗) *PAX4* LoF variant that was shown to be associated with increased risk of type 2 diabetes (T2D) in a single family [13]. The only reported family history of diabetes in either of our probands was in the maternal uncle of I-2, who was diagnosed with diabetes before the age of 30, is reported to be lean (BMI unknown), and is currently treated with a sulphonylurea. A DNA sample from this individual was not available for testing. The lack of a clear family history of diabetes in either family supports the finding from Laver et al. [19] that *PAX4* heterozygous variants are unlikely to be a cause of maturity-onset diabetes of the young (MODY) but may confer an increased risk of type 2 diabetes, as was found by Lau et al. [13].

### 2.2 PAX4 regulates pancreatic beta cell development and glucose sensitive insulin secretion

The identification of biallelic *PAX4* LoF variants as causing transient rather than permanent NDM is suggestive of a different role for PAX4 in human pancreatic development compared to that in mouse. In mice, homozygous *Pax4* knockout (KO) results in a near complete lack of mature pancreatic beta and delta cells and a significantly elevated alpha cell count compared to wildtype mice [9,12]. This in turn results in an inability of the murine pancreas to produce insulin or somatostatin, while glucagon production is significantly increased. *Pax4* KO mice are therefore born with diabetes and die shortly after birth. In contrast, the period of diabetes remission observed in our 2 patients with complete *PAX4* loss indicates that insulin-producing beta cells do still develop and are present in sufficient numbers for full glycaemic control during the remission period. Thus, our study provides another example of the differences in pancreatic development that exist between mice and humans [1]. Despite this, the observed transient remission suggests that PAX4 may contribute to beta cell proliferation, mirroring the ability of PAX4-expressing beta cells in mice to expand during pregnancy and periods of insulin resistance, and implying that certain aspects of PAX4-driven beta cell maintenance are conserved between species [20].

**Figure 2:**
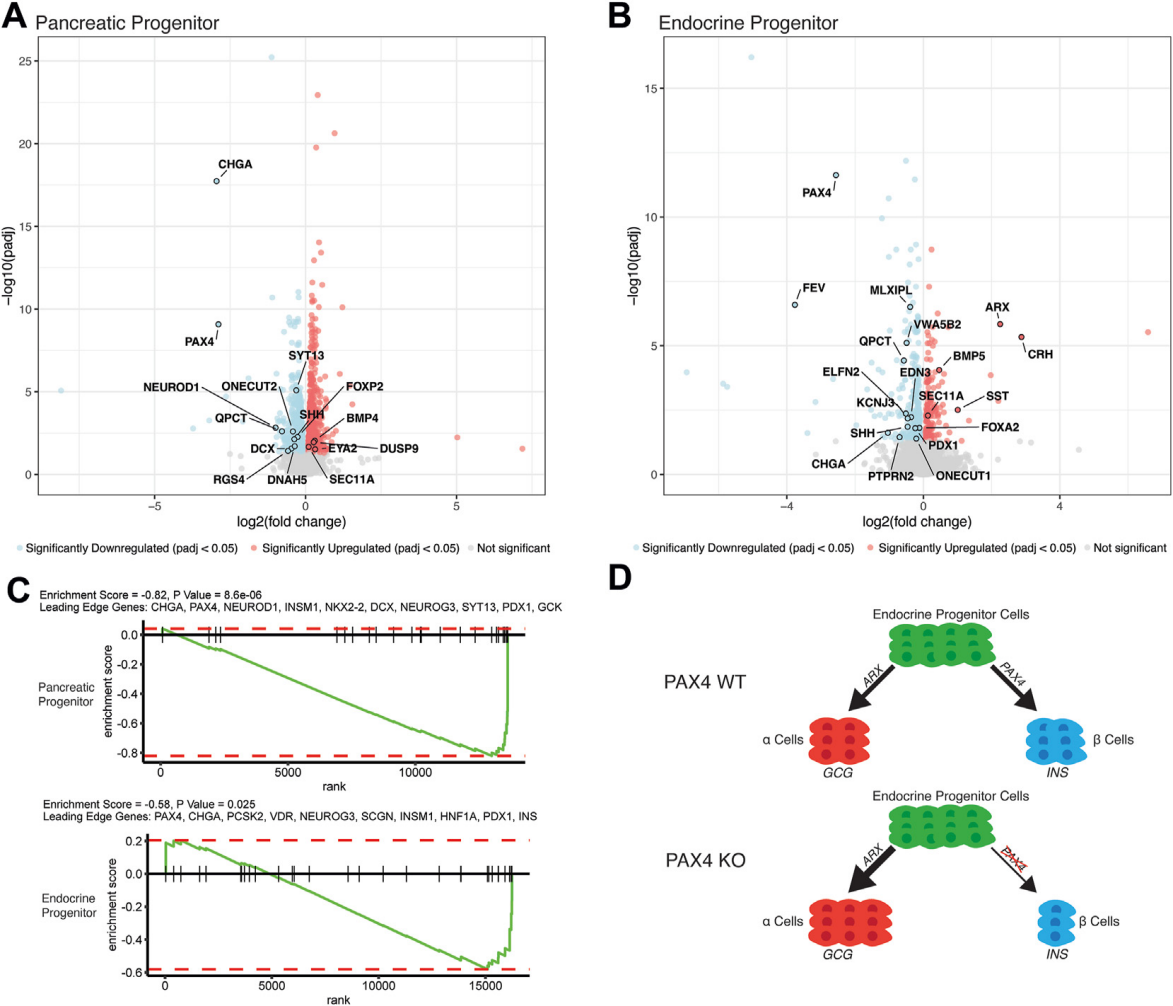
A) 1,529 genes were significantly differently expressed in S4 (Pancreatic Progenitor) *PAX4* Knockout (KO) cells compared to wildtype (WT) (757 downregulated and 772 upregulated). Significantly differently expressed genes overlapping with the Muraro [44] and Descartes [45] endocrine pancreatic cell type gene sets in MSigDB [27] are highlighted. B) 514 genes were significantly differently expressed in S5 (Endocrine Progenitor) *PAX4* KO cells compared to WT (324 downregulated and 190 upregulated). Significantly differently expressed genes overlapping with the Muraro [44] and Descartes [45] endocrine pancreatic cell type gene sets in MSigDB [27] are highlighted. C) Gene set enrichment analysis using the log2 fold change values for differently expressed genes at both the S4 and S5 stages against the hallmark pancreas beta cell gene set from MSigDB [27,28] revealed significant negative enrichment of beta cell gene expression in the *PAX4* KO compared to WT at both stages. Leading edge genes for each result are included. D) Proposed model for how loss of PAX4 disrupts human islet development.

Previously, Lau et al. performed RNA-seq on a biallelic *PAX4* knockout in a human iPSC-derived islet developmental model to investigate PAX4’s role in beta cell function and how *PAX4* haploinsufficiency elevates type 2 diabetes risk [13]. Loss of *PAX4* was shown to result in a bihormonal expression profile in stem cell derived islet cells, with increased expression of *GCG*, *SST* and *GHRL*. Additionally, there was a significant de-repression of alpha cell genes and reduction in expression of genes related to beta cell maturation, consistent with a loss of beta cell identity. To further explore how complete loss of PAX4 disrupts beta cell development and thus causes NDM, we re-interrogated this data with a focus on the role of PAX4 at earlier stages of development (pancreatic progenitors and endocrine progenitors). At the pancreatic progenitor stage, we saw a significant down regulation in genes with previously described roles in the specification of mammalian pancreatic endocrine cell fate, including *NEUROD1*, *FOXP2* and *ONECUT2* [[21], [22], [23]] in the *PAX4* KO (Figure 2A). This pattern continued into the endocrine progenitor cell stage where we saw a significant reduction in expression of the endocrine cell fate genes *FOXA2* and *ONECUT1* [23,24], but with the additional downregulation of *PDX1*, a gene that is critical to the maintenance of beta cell identity [25], and large upregulation of *ARX*, one of the primary drivers of alpha cell fate specification [26] (Figure 2B). We then performed gene set enrichment analysis of the differentially expressed genes at each stage against the hallmark pancreas beta cell gene set from MSigDB [27,28] and found that in both there was significant negative enrichment of pancreatic beta cell genes in the *PAX4* knockout cells (Figure 2C). Overall, this evidence suggests that, similarly to observations in mouse models [9,12], human *PAX4* expression is important for endocrine cell development and has a key role in directing progenitor cells toward a beta cell fate (Figure 2D).

**Figure 3:**
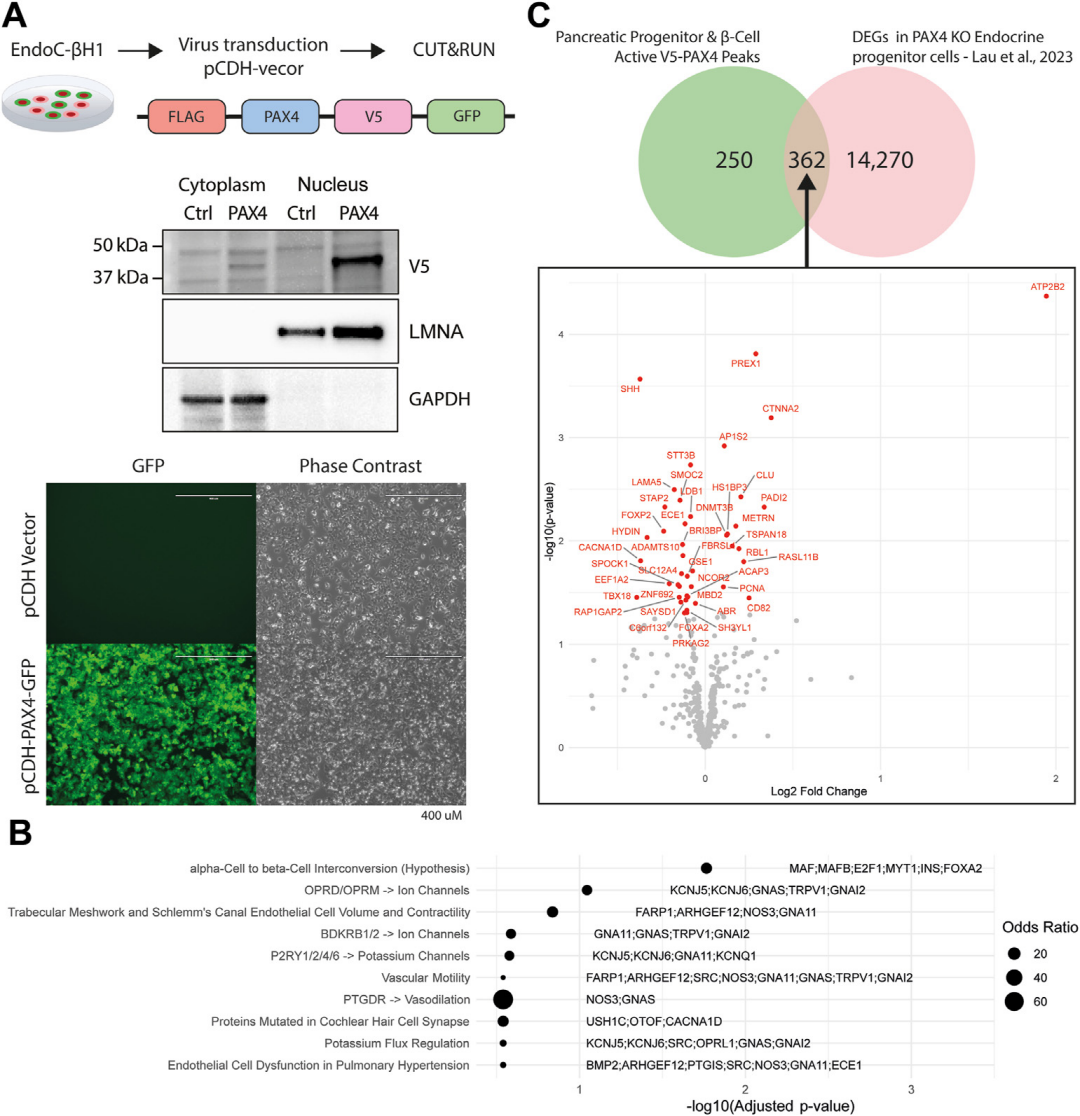
A) Cleavage Under Targets and Release Using Nuclease (CUT&RUN) sequencing of PAX4 binding targets in EndoC-βH1 cells. FLAG-PAX4-V5-GFP was introduced to the cells via lentiviral transduction, with bound regions then sequenced across 5 million reads. Western blotting and fluorescent microscope images confirmed nuclear localization and the overexpression of PAX4 in the EndoC-βH1 cells, respectively. B) 1,673 peaks were identified through analysis with SEACR [30], 612 of which overlapped regions of accessible chromatin in pancreatic progenitors and adult donor beta cells. Gene ontology enrichment analysis revealed a significant enrichment for genes belonging to a hypothesized alpha cell to beta cell interconversion pathway in the Elsevier Pathway Collection among the genes putatively regulated by the 612 accessible PAX4 peaks. C) We compared the genes bound by PAX4 with those that were dysregulated in the Lau et al. [13] *PAX4* knockout (KO) in endocrine progenitors, with a total of 362 overlapping genes, 43 of which were significantly differentially expressed in the KO cells compared to the wild-type.

To identify direct targets of PAX4 and further explore its role in human beta cell development and function, we performed a CUT&RUN [6] assay to identify PAX4-bound regions in the EndoC-βH1 human pancreatic beta cell model [29]. Due to the unavailability of ChIP-grade PAX4 antibodies, we introduced FLAG-PAX4-V5-GFP via lentiviral transduction and sequenced 5 million reads from PAX4-V5 antibody-bound regions (Figure 3A). Analysis using SEACR [30] identified 1,673 binding peaks (Supplemental Table 2). We then compared these peaks with chromatin accessibility data in adult donor beta cells from Chiou et al. [31] and embryonic stem cell-derived pancreatic progenitor cells from Geusz et al. [32]. Of the 1,673 CUT&RUN peaks, 612 overlapped with ATAC-seq peaks in these datasets, indicating that they are likely biologically relevant to beta cell development and function (Supplemental Table 3).

Gene ontology analysis of the genes proximal to the 612 beta cell- and pancreatic progenitor-accessible PAX4 binding peaks in the Elsevier Pathway Collection revealed an enrichment of genes in a hypothesized alpha-cell to beta-cell interconversion pathway (Figure 3B). This was the most significantly enriched pathway in the dataset and the only pathway that was still significant after adjustment for multiple testing (adjusted *P* = 0.017). The enrichment was driven by the presence of peaks overlapping promoters and enhancers of the genes *MAF*, *MAFB*, *E2F1*, *MYT1*, *INS* and *FOXA2*. The binding of PAX4 to the *INS* gene promoter is direct evidence for the importance of the gene in insulin secretion, while MAFB and FOXA2 are known to have essential roles in islet development and beta cell fate specification from human genetic studies [24,33]. MAF is a known regulator of alpha cell-specific genes and acts cooperatively with PAX6 to transactivate the glucagon promoter [34,35]. E2F1 and MYT1 have documented roles in beta cell proliferation [36,37]. Interestingly, many of the other nominally significantly enriched pathways identified in the analysis were related to potassium ion flux regulation. Potassium ion transportation is vital to insulin secretion [38]; pathogenic activating variants in the *ABCC8* and *KCNJ11* ATP-sensitive potassium channel subunit encoding genes are a common cause of both permanent NDM (PNDM) and TNDM [17,39].

Comparison of the 612 beta cell- and pancreatic progenitor-accessible peaks with previously reported differentially expressed genes (DEGs) in *PAX4* KO stem cell derived endocrine progenitor cells from Lau et al. revealed an overlap of 362 genes, 43 of which showed significant differences in expression between the wild-type and KO cells (Figure 3C). The overlap included *FOXA2*, which has a well-established essential role in pancreatic islet development and function [24], and *FOXP2* and *SHH*, which prior mammalian studies suggest may also play important roles in these processes [22,40]. The overlap also included the *CACNA1D* gene, which encodes a subunit of the Cav1.3 voltage-gated calcium channel that forms part of the glucose-sensitive insulin release pathway [41]. Autosomal dominant activating variants in *CACNA1D* are a known cause of congenital hyperinsulinism, a disease characterized by unregulated insulin secretion from pancreatic beta cells [42]. We also examined whether the PAX4 binding sites identified from the CUT&RUN data are enriched in T2D GWAS loci [43]; however, no significant enrichment was observed. Overall, the analysis of the PAX4 CUT&RUN data shows a clear role of the gene in human islet development and in the regulation of genes important for glucose-sensitive insulin secretion.

## 3 Conclusion

Our study establishes bi-allelic *PAX4* LoF variants as a novel genetic cause of NDM. The transient diabetes phenotype observed in our patients suggests that beta cells can develop in humans in the absence of PAX4. This differs from observations in rodent *Pax4* KO models that have complete loss of beta cells resulting in permanent neonatal diabetes and postnatal death. Functional profiling of *PAX4* in human pancreatic cell models using CUT&RUN and RNA-Seq data highlights this gene’s regulatory role for PAX4 in both islet development and glucose-sensitive insulin secretion. This highlights a specific but non-essential role in human beta-cell development and provides functional evidence for its mechanism not only in monogenic disease but also as a risk gene for type 2 diabetes.

## CRediT authorship contribution statement

**James Russ-Silsby:** Writing – review & editing, Writing – original draft, Visualization, Validation, Software, Project administration, Methodology, Investigation, Funding acquisition, Formal analysis, Data curation, Conceptualization. **Yunkyeong Lee:** Writing – review & editing, Writing – original draft, Visualization, Validation, Project administration, Methodology, Investigation, Formal analysis, Data curation, Conceptualization. **Varsha Rajesh:** Writing – review & editing, Visualization, Investigation, Formal analysis. **Mahsa Amoli:** Writing – review & editing, Resources, Data curation. **Nasser Ali Mirhosseini:** Writing – review & editing, Resources, Data curation. **Tushar Godbole:** Writing – review & editing, Resources, Data curation. **Matthew B. Johnson:** Writing – review & editing, Resources. **D. Evelyn Ibarra:** Writing – review & editing, Methodology. **Han Sun:** Writing – review & editing, Writing – original draft, Visualization, Software, Methodology, Formal analysis, Data curation. **Nicole A.J. Krentz:** Writing – review & editing, Methodology, Conceptualization. **Matthew N. Wakeling:** Writing – review & editing, Software, Methodology, Data curation. **Sarah E. Flanagan:** Writing – review & editing, Resources. **Andrew T. Hattersley:** Writing – review & editing, Resources. **Anna L. Gloyn:** Writing – review & editing, Writing – original draft, Validation, Supervision, Resources, Project administration, Methodology, Funding acquisition, Conceptualization. **Elisa De Franco:** Writing – review & editing, Writing – original draft, Validation, Supervision, Resources, Project administration, Methodology, Funding acquisition, Conceptualization.

## Declaration of competing interest

The authors declare the following financial interests/personal relationships which may be considered as potential competing interests: Anna L. Gloyn’s spouse is an employee of Genentech and holds stock options in Roche. The other authors declare no competing interests. If there are other authors, they declare that they have no known competing financial interests or personal relationships that could have appeared to influence the work reported in this paper.

## Figures and Tables

**Table:** Key Resources Table

REAGENT or RESOURCE	SOURCE	IDENTIFIER
**Antibodies**		
Rabbit monoclonal Anti-H3K4me3 antibody for CUT&RUN	EpiCypher	Cat# 13-0041, RRID: AB_3076423
Rabbit polyclonal Anti-IgG negative control antibody for CUT&RUN and CUT&Tag	EpiCypher	Cat# 13-0042, RRID: AB_2923178
Mouse monoclonal Anti-V5 antibody	Abcam	Cat# ab27671, RRID: AB_471093
Mouse monoclonal anti-lamin A/C antibody	Cell signaling technology	Cat# 4777, RRID: AB_10545756
Rabbit polyclonal Anti-GAPDH antibody	Abcam	Cat# ab37168, RRID: AB_732652
**Chemicals, peptides, and recombinant proteins**		
jetPRIME transfection reagent	Polyplus	Cat# 101000046
Puromycin	Gibco	Cat# A1113803
Tris pH 7.4	Millipore	Cat# 648315
Sodium chloride	Sigma–Aldrich	Cat# 31434
Triton X-100	ThermoFisher scientific	Cat# 85111
Sodium deoxycholate	Sigma–Aldrich	Cat# D6750
Sodium dodecyl sulfate	Sigma–Aldrich	Cat# L3771
Protease inhibitor cocktail	Roche	Cat# 11697498001
4x Laemmli sample buffer	Bio-rad	Cat# 1610747
β-mercaptoethanol	Bio-rad	Cat# 1610710
Tris-glycine-SDS buffer	Bio-rad	Cat# 1610772EDU
Nonfat dry milk powder	Research products international	Cat# M17200–500.0
Phosphate buffered saline	Sigma–Aldrich	Cat# P4417
Tween-20	Sigma–Aldrich	Cat# P1379
DMEM, low glucose	Gibco	Cat# 11885084
DMEM, high glucose	Gibco	Cat# 11965092
Bovine serum albumin fraction V, heat shock, fatty acid free	Roche	Cat# 3117057001
Nicotinamide	Sigma–Aldrich	Cat# N3376
GlutaMAX™	Gibco	Cat# 35050061
Transferrin	Sigma–Aldrich	Cat# T8158
Sodium selenite	Sigma–Aldrich	Cat# S5261
Fetal bovine serum, heat inactivated	Gibco	Cat# 10438026
10,000U/mL penicillin/10,000ug/ml streptomycin	Gibco	Cat# 15140122
0.05% Trypsin–EDTA	Gibco	Cat# 25300062
Bovine fibronectin	Sigma Aldrich	Cat# F1141
ECM gel	Sigma Aldrich	Cat# E1270
mTeSR™1 basal medium	Stem cell technologies	Cat# 85850
Y-27632	Stem cell technologies	Cat# 72304
MCDB131 media	Corning	Cat# MT15100CV
Sodium bicarbonate	Sigma–Aldrich	Cat# S5761
Matrigel hESC-Qualified matrix	Corning	Cat# 354277
Matrigel matrix basement membrane growth factor reduced	Corning	Cat# 356230
Glucose	Sigma–Aldrich	Cat# G7021
Accutase	Stem cell technologies	Cat# 7920
Activin A	ThermoFisher scientific	Cat# 12014E; hiPSC differentiation stage 1
CHIR99021	Stem cell technologies	Cat# 72054; hiPSC differentiation stage 1
KGF (FGF-7)	Gibco	Cat#AF-100-19; hiPSC differentiation stage 2, 3 & 4
Ascorbic acid	Sigma–Aldrich	Cat# A4544; hiPSC differentiation stage 2, 3 & 4
Retinoic acid	Millipore sigma	Cat# R2625; hiPSC differentiation stage 3 & 4
SANT-1	Millipore sigma	Cat# S4572; hiPSC differentiation stage 3 & 4
LDN-193189	Reprocell	Cat# 04-0074; hiPSC differentiation stage 3 & 4
TPPB	Biotechne	Cat# 5343; hiPSC differentiation stage 3 & 4
ITS-X	ThermoFisher scientific	Cat# 41400045; hiPSC differentiation stage 3 & 4
BsaI restriction enzyme	New England biolabs	Cat# R3733S
**Critical commercial assays**		
MycoAlert™ plus mycoplasma detection kit	Lonza bioscience	Cat# LT07-710
Nuclear and cytoplasmic extraction kit	ThermoFisher scientific	Cat# 78833
Pierce™ BCA protein assay kit	ThermoFisher scientific	Cat# 23227
Criterion TGX stain-free precast gel	Bio-rad	Cat# 5678094
Trans-blot turbo midi 0.2 uM PVDF transfer packs	Bio-rad	Cat# 1704157
CUTANA™ ChIC/CUT&RUN assay kit	EpiCypher	Cat# 14-1048
CUTANA™ CUT&RUN library prep kit	EpiCypher	Cat# 14-1001
RNA isolation kit	Sigma–Aldrich	Cat# 83913
**Deposited data**		
Raw PAX4 CUT&RUN data	This study	EGA: EGAS50000000857
Raw PAX KO and WT hiPSC-derived islet cell RNA-Seq data	Lau et al. [13]	EGA: EGAS00001006036
**Experimental models: Cell lines**		
Human: SB Ad3.1 hiPSC line	Lonza	Cat# CC-2511, tissue acquisition number: 23447
Human: EndoC-βH1	Univercell biosolutions	RRID: CVCL_L909
Human: HEK293T	Invitrogen	RRID: CVCL_0063
**Oligonucleotides**		
PAX4 R126∗ forward primer	Integrated DNA technologies	5′-TTTGTGCTGAAGGGCTTTGC-3′
PAX4 R126∗ reverse primer	Integrated DNA technologies	3′-TTGGGTTGTTGTGAGGGTGA-5′
PAX4 wildtype allele probe	Integrated DNA technologies	5′-CAGGACTCGGTTGATG-3′
PAX4 mutant (R126∗) allele probe	Integrated DNA technologies	5′-CAGGACTCAGTTGATG-3′
**Recombinant DNA**		
pCDH plasmid		Gift from Dr. Adrian Kee Keong Teo
pCDH-FLAG-PAX4-V5-GFP plasmid		Gift from Dr. Adrian Kee Keong Teo
pMD2.G plasmid	Addgene	Cat# 12259, RRID:Addgene_12259
psPAX2 plasmid	Addgene	Cat# 12260, RRID:Addgene_12260
**Software and algorithms**		
Genome analysis toolkit (GATK) v3.7	Broad institute	https://software.broadinstitute.org/gatk; RRID:SCR_001876
nf-core CUT&RUN pipeline v3.2.2	Cheshire et al. [46]	https://nf-co.re/cutandrun/3.2.2
SEACR v1.3	Meers et al.	https://github.com/FredHutch/SEACR
R v4.4.0	R core team	http://www.r-project.org; RRID:SCR_001905
enrichR	Kuleshov et al. [47]	https://github.com/wjawaid/enrichR; RRID:SCR_001575
MEME suite v5.5.7	Bailey et al. [48]	http://meme-suite.org; RRID:SCR_001783
Bedtools v2.30.0	Quinlan laboratory	https://github.com/arq5x/bedtools2; RRID:SCR_006646
STAR v2.7.11b	Dobin et al. [49]	http://code.google.com/p/rna-star; RRID:SCR_004463
RSEM v1.33	Li and Dewey. [50]	https://github.com/deweylab/RSEM
RUVSeq v1.42.0	Risso et al. [51]	https://bioconductor.org/packages/release/bioc/html/RUVSeq.html
DESeq2	Love et al. [52]	https://bioconductor.org/packages/release/bioc/html/DESeq2.html; RRID:SCR_015687
FGSEA v1.34.0	Korotkevich et al. [53]	https://bioconductor.org/packages/release/bioc/html/fgsea.html
**Other**		
Neon Electroporation system	Invitrogen	Cat# 10431915
EVOS FL imaging system	Invitrogen	Cat# AMF4300
Criterion™ cell and PowerPac™ basic power supply	Bio-rad	Cat# 1656019
Trans-blot turbo transfer system	Bio-rad	Cat# 1704150
ChemiDoc MP imaging system	Bio-rad	Cat# 12003154

## Data Availability

Data will be made available on request.

## Experimental model and study participant details

### Study Participants

Individuals with clinically suspected monogenic diabetes were recruited by their clinicians for molecular genetic analysis in the Exeter Molecular Genetics Laboratory. Consanguineous individuals had either reported parental relatedness at referral or had homozygous segments making up more than 3% of their genome. The study was conducted in accordance with the Declaration of Helsinki, and all subjects or their parents gave informed consent for genetic testing with ethical approval received from the Genetic Βeta-cell Research Bank, Exeter, U.K.

### Human Induced Pluripotent Stem Cells (hiPSCs)-Derived Pancreatic Endoderm (PE) Cells

hiPSCs were cultured on hESC-qualified matrigel (Corning, 354277)-coated plates in mTeSR™1 basal medium (STEMCELL™ Technologies, 85850) supplemented with 1% Penicillin/Streptomycin (Gibco, 15140122) and 10 uM ROCK inhibitor (Y-27632, STEMCELL™ Technologies, 72304) with daily media changes and passaged using Accutase (STEMCELL™ Technologies, 7920). A 200 nucleotide single-stranded oligodeoxynucleotide repair template (purchased from Integrated DNA Technologies) was designed containing the p.(Arg126∗) (Chr7(GRCh37):g.127254596G > A) variant. A silent mutation was also included at codon 126 to remove BsaI/BseRI restriction enzyme sites for genotyping. 1 × 10^6^ NKX6.1-GFP hiPSCs [54] were electroporated with an RNP complex (gRNA, Alt-R Cas9 enzyme) and Alt-R HDR donor oligo using the Neon Electroporation System (Invitrogen, 10431915). Electroporate using following parameters: 1,200 V, 30 ms, 1 pulse. Single-cell colonies were picked, expanded and screened using the BsaI restriction enzyme (New England Biolabs, R3733S). All clones (p.(Arg126∗) WT sham, Het and Hom) were further validated by Sanger sequencing using the 5′-TTTGTGCTGAAGGGCTTTGC-3′ (forward) and 3′-TTGGGTTGTTGTGAGGGTGA-5′ (reverse) primer sequences. CRISPR genome edited hiPSCs were seeded on Growth Factor Reduced Basement Membrane Matrigel (Corning, 356230)-coated plates in mTeSR™1 basal medium supplemented with 10 uM ROCK inhibitor. The differentiation was started following day of seeding in MCBD131 media (Corning, MT15100CV) supplemented 1% Penicillin/Streptomycin, sodium bicarbonate (Sigma-aldrich, S5761), GlutaMAX™ (Gibco, 35050061) and glucose (Sigma-aldrich, G7021) and proceeded through a seven-stage differentiation protocol (stages 1–4; stage 1: definitive endoderm (DE), stage 2: primitive gut tube (PGT), stage 3: posterior foregut (PFG) and stage 4: pancreatic endoderm (PE)) [15]. HiPSC-derived PE cells were treated with 100 μg/ml cycloheximide (CHX, sigma-aldrich, C4859) or DMSO as a negative control for 4 h to inhibit NMD. Following the treatment, total RNA was isolated and cDNA was synthesized for *PAX4* transcript analysis by qPCR.

### EndoC-βH1 Cells

EndoC-βH1 cells were cultured in low glucose DMEM (Gibco, 11885084) supplemented with 2% BSA (Roche, 3117057001), 10 mM nicotinamide (Sigma–Aldrich, N3376), 2 mM GlutaMAX™ (Gibco, 35050061), 50 μM β-mercaptoethanol (Bio-Rad, 1610710), 5.5 μg/mL transferrin (Sigma–Aldrich, T8158), 6.6 ng/mL sodium selenite (Sigma–Aldrich, S5261) and 1% Penicillin/Streptomycin (Gibco, 15140122) in tissue culture plates precoated with high glucose DMEM (Gibco, 11965092) supplemented with 2 μg/mL bovine fibronectin (Sigma–Aldrich, F1141) and 1% ECM (Sigma–Aldrich, E1270) in a 5% CO_2_ humidified incubator at 37 °C. Cells were passaged weekly with 0.05% Trypsin/EDTA (Gibco, 25300062).

### HEK293T Cells

HEK293T cells were cultured in high glucose DMEM (Gibco, 11965092) supplemented with 10% Fetal Bovine Serum (Gibco, 10438026) and 1% Penicillin/Streptomycin (Gibco, 15140122). Cells were passaged twice a week with 0.05% Trypsin/EDTA. All mammalian cells were regularly tested to be mycoplasma free using a MycoAlert™ Plus mycoplasma detection kit (Lonza Bioscience, LT07-710).

## Method Details

### Genome Sequencing

DNA was extracted from peripheral blood mononuclear cells and saliva samples and sequenced using an Illumina HiSeq 2500, an Illumina HiSeq X10 and a BGI-Seq 500, producing 100bp and 150bp paired-end reads. Genome sequencing data was then processed according to the GATK best-practice workflow for genome sequencing data (https://gatk.broadinstitute.org).

### Targeted Next Generation Sequencing

Target regions were captured and enriched using Agilent SureSelect custom capture and Twist Bioscience Custom panel kits. These regions were then sequenced using an Illumina NextSeq or an Illumina NovaSeq X, producing 150bp paired-end reads. The sequencing data was processed using a GATK best-practice workflow optimized for targeted and exome sequencing (https://gatk.broadinstitute.org).

### Allele-Specific qPCR of CRISPR Edited Human Induced Pluripotent Stem Cell (iPSC)-Derived Pancreatic Endoderm Cells

Pancreatic endoderm samples were lysed and RNA extracted using RNA isolation kit (Sigma–Aldrich, 83913). RNA was treated with DNase to remove any genomic DNA and quality and concentration determined on Nanodrop. mRNA was reverse transcribed to cDNA. cDNA was then incubated with a SNP genotyping assay containing two probes that separately bound the WT and stop-gain p.(Arg126∗) *PAX4* alleles. Both probes were coupled with unique fluorophores to separately track amplification of the 2 alleles with each round of PCR (HEX and FAM).

### CUT&RUN Assay, Library Preparation and Sequencing in EndoC-βH1 cells

CUT&RUN was performed on nuclei extracts from FLAG-PAX4-V5-GFP overexpressing EndoC-βH1 cells using CUTANA™ ChIC/CUT&RUN Kit version 2.1 (EpiCypher, 14–1048) according to the manufacturer’s instructions. Briefly, samples were incubated with 0.5 ug of mouse anti-V5 tag (Abcam, ab27671), H3K4me3 positive control (EpiCypher, 13–0041) and rabbit anti-IgG negative control (EpiCypher, 13–0042) antibodies. Library preparation was performed using the CUTANA™ CUT&RUN Library Prep Kit (version 1.2, EpiCypher, 14–1001). Libraries were sequenced using Novaseq 5M paired end reads per sample.

### Lentiviral Transduction and Overexpression in EndoC-βH1

pCDH empty and pCDH-FLAG-PAX4-V5-GFP plasmids [13] were co-transfected in HEK293T with packaging vectors pMD2.G (Addgene, 12259) and psPAX2 (Addgene, 12260) using jetPRIME transfection reagent (Polyplus, 101000046). Viral supernatant was collected at 72h post-transfection and EndoC-βH1 cells were transduced overnight and selected for 7d in 4 μg/ml puromycin (Gibco, A1113803) with media changes as required. Overexpression of the GFP including construct was confirmed using the EVOS FL Imaging System (Invitrogen, AMF4300).

### Nuclear and Cytoplasmic Extraction and Western Blot Analysis

Nuclear and Cytoplasmic Extraction Kit (ThermoFisher, 78833) was used for cell lysis and extraction of separate nuclear and cytoplasmic protein fractions according to the manufacturer’s specifications. For SDS-PAGE, whole cell protein lysates were obtained from cell pellets through lysis in RIPA buffer (50 mM Tris pH 7.4 (Millipore, 648315), 150 mM sodium chloride (Sigma–Aldrich, 31434), 1% Triton X-100 (ThermoFisher Scientific, 85111), 0.5% sodium deoxycholate (Sigma–Aldrich, D6750), 0.1% sodium dodecyl sulfate (Sigma–Aldrich, L3771)) containing 1x protease inhibitor cocktail (Roche, 11697498001). Protein was quantified using Pierce™ BCA Protein Assay Kit (ThermoFisher Scientific, 23227). 15 ug of total protein in 4x Laemmli sample buffer (Bio-Rad, 1610747) containing 10% β-mercaptoethanol (Bio-Rad, 1610710), and boiled for 5 min at 100 °C. Denatured samples were separated on 4–20% Criterion™ TGX Stain-Free Precast Gel (Bio-Rad, 5678094) in Tris-glycine-SDS buffer (Bio-Rad, 1610772EDU) using Criterion™ Cell and PowerPac™ Basic Power Supply (Bio-Rad, 1656019) and the gel was transferred onto a 0.2 μM polyvinylidene difluoride (PVDF, Bio-Rad, 1704157) membrane using the Trans-Blot Turbo Transfer System (Bio-Rad, 1704150). Nonspecific antibody binding was blocked in 5% dry milk powder (Research Products International, M17200–500.0) in 1x phosphate buffered saline (Sigma–Aldrich, P4417) containing 0.1% Tween-20 (Sigma–Aldrich, P1379) (PBST) and the membranes were incubated with primary antibodies (V5 (Abcam, ab27671), LMNA (Cell Signaling Technology, 4777) and GAPDH (Abcam, ab37168)) at 4 °C overnight and secondary antibodies at room temperature for 1h. Blots were acquired and quantified using the ChemiDoc MP Imaging System (Bio-Rad, 12003154).

## Quantification and statistical analysis

### Transcriptomic Analysis in Stem Cell-Derived Islets

RNA-Seq data for PAX4 knockout (KO) and corresponding wildtype (WT) induced pluripotent stem cell (iPSC) derived pancreatic progenitor and endocrine progenitor cells from Lau et al. [13] (protocol B) was aligned to the GRCh38 reference using STAR [49] and quantified at the transcript level using RSEM [50]. Count matrices were then normalised and analysed using DESEQ2 [52]. Additionally, RUVr [51] (*k* = 6) was used to reduce the impact of unwanted variation on the differential expression analysis. Differentially expressed genes were compared with Muraro [44] and Descartes [45] endocrine pancreatic cell gene sets from the C8 collection on MSigDB [27] to identify functionally relevant genes to be highlighted in differential expression plots. Gene set enrichment analysis was performed using the Fast Gene Set Enrichment (FGSEA) [53] package in R against the hallmark pancreas beta cell gene set from MSigDB [27,28].

### CUT&RUN Analysis

CUT&RUN Fastq files from the CUT&RUN experiments were processed following the nf-core CUT&RUN pipeline (https://nf-co.re/cutandrun/3.2.2/) [46]. Peaks were called using the SEACR peak caller under the stringent parameter [30]**.** ATAC-Seq peaks in adult donor beta cells from Chiou et al. [31] and pancreatic progenitor cells from Geusz et al. [32] were used to prioritize binding sites that were likely to be functionally relevant. Gene ontology analysis was performed using EnrichR (https://github.com/wjawaid/enrichR) [47] in R based on the Elsevier Pathway Collection. To assess whether the PAX4 binding sites identified from the CUT&RUN data are enriched in T2D GWAS loci, we downloaded the credible sets from the Mahajan et al. [43] multi-ancestry genetic study, converted the coordinates from hg19 to hg38, and performed a Fisher’s exact test using bedtools (v2.30.0). Find Individual Motif Occurrences (FIMO) and Multiple Em for Motif Elicitation (MEME) from the MEME Suite v5.5.7 [48] were also used to examine motif enrichment within the CUT&RUN peaks (Supplemental Table 4, Supplemental Fig. 1).
