# Supplementary material for: Complete loss of PAX4 causes transient neonatal diabetes in humans
Source: Mol Metab. 2025 Jul 2;99:102201. doi: 10.1016/j.molmet.2025.102201 (PMC12275979; doi:10.1016/j.molmet.2025.102201)
Supplement: Multimedia component 1 [file mmc1.pdf]

Supplementary Figures

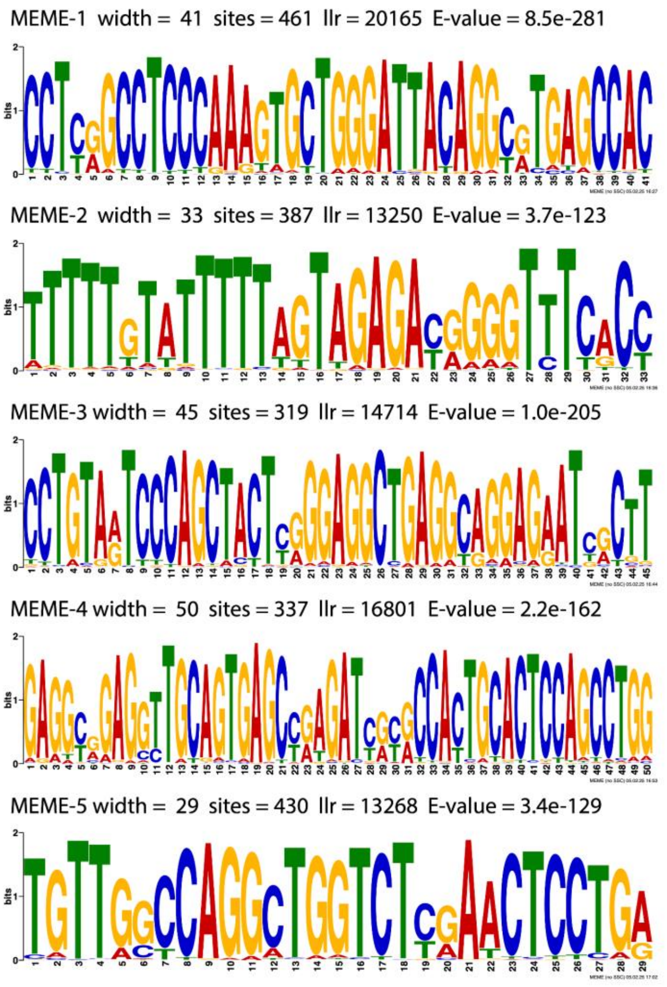

Supplementary figure 1: Motifs discovered using MEME on the PAX4 cut-and-run peaks. Multiple Em for Motif Elicitation (MEME) from the MEME Suite (v5.5.7) was used to discover novel motifs from the genomic sequences of the peaks identified in the CUT&RUN data. The top 5 motifs discovered are reported here.

Supplemental Tables

Supplemental table 1: Clinical features of patients with biallelic *PAX4* variants

	Individual 1 (I-1)	Individual 2 (I-2)
**Homozygous *PAX4* Variant**	p.(Arg126*)	c.-352_104del
**Birth weight**	2400g @ 36wks (-1.16SD)	2200g @ 38wks (-2.98 SD)
**Diagnosis Age**	45 days (diabetic ketoacidosis)	9 weeks (diabetic ketoacidosis)
**Remission Age**	7 months	8 months
**Relapse Age**	2.4 years	6.7 years
**Current treatment**	Basal Bolus Insulin (1U/Kg/Day) + Glibenclamide	Basal Bolus Insulin (Dose Unkown)
**Additional features**	Learning Disorder	
**Referral Country**	Iran	India
**Random non-fasting C-peptide (Age)**	Unknown	1ng/ml (5 Years)
**Most Recent HbA1c (Age)**	53 mmol/mol (12 Years)	79 mmol/mol (6 Years)
